# Complete Kawasaki disease after COVID-19 infection in an infant

**DOI:** 10.12669/pjms.39.2.6359

**Published:** 2023

**Authors:** Maliha Tahir, Ramlah Mehmood, Mirza Sultan Ahmad

**Affiliations:** 1 Dr. Maliha Tahir, MBBS., King Edward Medical, University, Lahore, Pakistan. Medical Officer Pediatric, Department of Paediatrics Fazl-e-Omar Hospital, Chenab Nagar, District Chiniot, Pakistan; 2 Dr. Ramlah Mehmood, MBBS, FCPS (Pediatrics)., Consultant Pediatrician, Department of Paediatrics Fazl-e-Omar Hospital, Chenab Nagar, District Chiniot, Pakistan; 3 Dr. Mirza Sultan Ahmad, MBBS, DCH, FCPS (Pediatrics)., Head of Department, Pediatrics, Department of Paediatrics Fazl-e-Omar Hospital, Chenab Nagar, District Chiniot, Pakistan

**Keywords:** Kawasaki Disease, Covid-19, Vasculitis, Infants, Developing Country

## Abstract

Kawasaki disease is a generalized systemic vasculitis that affects blood vessels throughout the body. The aetiology of Kawasaki disease is still unknown but is thought to be related to the combined effects of the immune response, genetic susceptibility, and infections including COVID-19. In this case report, we present a seven months old male infant who presented to us with fever, swollen lips, ulcers in the mouth, enlarged tonsils, strawberry tongue, conjunctivitis and generalised non-blanchable maculopapular rash. The detailed workup fulfilled the criteria of Kawasaki disease. The COVID-19 IgM antibodies were positive. The patient was treated with IV Immunoglobulins, IV methylprednisolone and Aspirin. The repeat echocardiography on six weeks follow-up turned out normal. In conclusion, there should be a high index of suspicion of Kawasaki disease while evaluating pediatric patients with COVID-19 infection so that timely intervention can be made to prevent complications. Prospective studies are needed to evaluate the relationship between Kawasaki Disease and COVID-19 infection.

## INTRODUCTION

Kawasaki disease (KD) is an acute, self-limited, systemic vascular disease that affects mostly small and medium-sized blood vessels. It mostly occurs in children under five years of age of different ethnicities all around the world.^1^ Diagnostic criterion as outlined by American Heart Association in 2017, includes fever for five or more days, lips and oral mucosal changes, bilateral non-purulent conjunctival injection, polymorphous rash, peripheral extremity changes with subsequent desquamation of fingertips, and cervical lymphadenopathy of more than 1.5 cm in size.^2^ The aetiology of Kawasaki disease is still unknown but is thought to be related to combined effects of the immune response, genetic susceptibility, and infections.^3^ Viral respiratory infections, including SAR-CoV-2 (COVID-19), have been suggested as triggers for Kawasaki disease. It has been observed that in the context of SARS-CoV-2 circulation a greater proportion of patients with Kawasaki disease might present a severe phenotype with cardiac complications.^4^ In a study conducted at Shifa International Hospital Pakistan complete KD was present in 72% of the studied cohort of children with Kawasaki disease. Coronary artery abnormalities were present in one-third of these children at a younger age and more common in those with incomplete KD but had recovered in most.^5^ Here we present a case of a seven-month-old male infant with Kawasaki Disease and a concurrent diagnosis of COVID-19 infection.

## CASE REPORT

**Fig.1:**
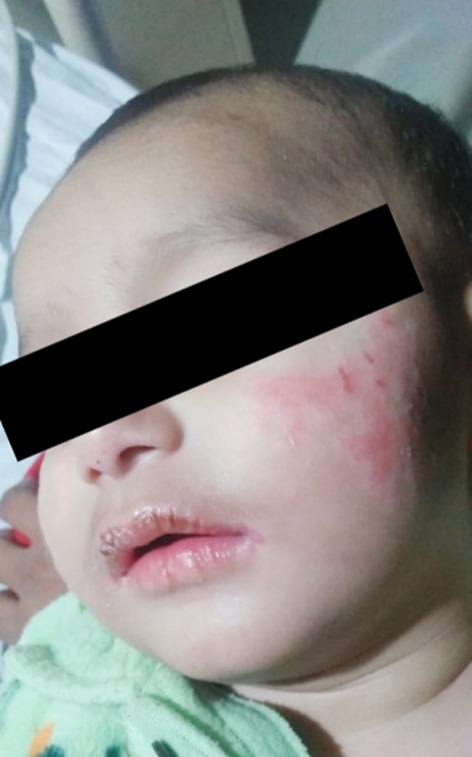
Rash in the resolving phase and cracked lips.

A seven-month-old male infant presented in our outpatient department with an acute history of high-grade fever, documented up to 102F along with rash for one day. On physical examination, the patient was irritable, with non pruritic non-blanchable generalised maculopapular rash, non-exudative conjunctivitis, swollen lips, ulcers in the mouth, enlarged hyperaemic tonsils, and strawberry tongue (Fig.1). There was hepatomegaly, firm non-tender up to 5cm but no lymphadenopathy. The patient was admitted to our Paediatric ward for further workup and management with a differential diagnosis of Staphylococcal scalded skin syndrome, Scarlet fever and Kawasaki disease. The patient was started on broad-spectrum antibiotics along with other supportive management and relevant workup was started.

Initial laboratory investigations showed, Hemoglobin (Hb) 10g/dl, leucocyte count (WBC) 13x10^9^/L with predominant neutrophils (72%) and normal platelet (PLT) count 281x10^9^/L. Quantitative C-reactive protein (CRP) was 144mg/dl, erythrocyte sedimentation rate (ESR) was 54mm/hr, Antistreptolysin O (ASO) titre was normal (<200), and urine routine showed 10-12 pus cells. COVID-19 Rapid antigen test was negative but the COVID antibody (IgM) was positive. Chest X-ray was normal. Other workup including coagulation profile, liver function tests, renal function tests, serum electrolytes and MP was unremarkable.

In the subsequent week, patient had persistent fever spikes along with the development of bilateral non-pitting pedal oedema. The repeat complete blood count (CBC) showed mild anaemia Hb 9.6g/dl, raised leukocyte count 27.5x10^9^/L and thrombocytosis with platelet count of 504x10^9^/L. Quantitative CRP and ESR were persistently high 135mg/dl and 82mm/hr respectively. The multisystem inflammatory syndrome in children (MIS-C) markers, serum ferritin 205ng/ml, LDH 321U/L and D-dimer 1061ng/ml were normal. Cardiac enzymes (Trop T <3pg/ml and ProBNP 257pg/ml) and ANA were negative. Throat swab culture grew streptococcus specie. The blood and urine cultures were negative. Ultrasound abdomen showed gall bladder wall oedema with normal liver echotexture. Diagnosis of complete Kawasaki disease was finalised.

**Fig.2:**
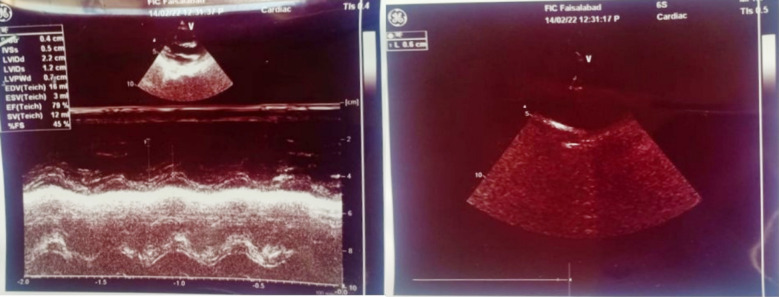
Dilated left coronary artery and right artery with no clot thrombus and good biventricular function.

Echocardiography was done that showed dilated left and right coronary arteries with good biventricular function. No clot, thrombus or pericardial effusion (Fig.2). The patient was initially treated with Intravenous methylprednisolone for three days (due to financial constraints), followed by Intravenous Immunoglobulins (IVIG) 2g/kg. Aspirin was also started.

Symptoms improved after steroid administration with complete resolution of fever after IVIG. Later periungual skin peeling was also observed. Patient was discharged on oral steroids and low dose Aspirin. The patient was advised to avoid live vaccines and called for a follow-up. Follow up visit after one week showed marked improvement in inflammatory markers. Repeat echocardiography after 06 weeks showed normal coronaries.

## DISCUSSION

Kawasaki disease is a generalized systemic vasculitis that affects blood vessels throughout the body. It is characterized by systemic inflammation in all the medium-sized arteries and in multiple organs and tissues during the acute febrile phase that can lead to hepatitis, interstitial pneumonitis, gastrointestinal abdominal pain, vomiting, diarrhea, gallbladder hydrops, aseptic meningitis, pyuria, and pancreatitis etc.^2^ The diagnosis of KD is often overlooked in febrile children in developing countries where infectious diseases are still prevalent. There are two forms of KD: complete and incomplete. Diagnosis of complete KD requires fever of at least five days’ duration along with four or five of the principal clinical features. Incomplete KD is diagnosed when a patient presents with fever for five days or longer, two or three of the principal clinical features, and laboratory findings suggestive of the disease or echocardiographic abnormalities.^6^

The patient in our case was an infant who presented with a rash and had coronary involvement whereas, a study conducted in Namazee tertiary referral center, Iran reported significantly higher skin rash in COVID-19 positive patients, but coronary involvement was more prevalent in COVID-19 negative patients. Moreover, a high percentage of KD patients were seen COVID-19 positive and with the age of onset lower for KD during the COVID-19 pandemic than before.^7^

There were no respiratory symptoms or comorbid conditions in our patient and the diagnosis of COVID-19 was done on the basis of positive antibodies. A study conducted at Children’s hospital Lahore Pakistan reported eight children fulfilling the criteria of multi-system inflammatory syndrome in children, all had positive antibodies whereas COVID PCR was positive in only three children, there were no classical respiratory symptoms or underlying comorbidities in all eight children.^8^

Incidence of children presenting with a severe inflammatory syndrome with KD-like features has also increased during the COVID-19 pandemic. The multisystemic inflammatory syndrome in children (MIS-C) related to the SARS-CoV-2 pandemic, also termed Kawasaki-like disease, appears to share clinical, pathogenetic and laboratory features with KD. In MISC, studies have reported resistance to IVIG infusion treatment.^9^ A case report from Karachi Pakistan has also reported resistance to IVIG in MISC.^10^ The patient in our case received both steroids and immunoglobulins though symptoms were completely subsided after immunoglobulins.

Updated AHA guidelines also include considering the use of adjuvant corticosteroid therapy in patients at high risk of IVIG resistance, and the change in steroid regimen for refractory KD to include both pulse-dose IV methyl prednisone and longer course of prednisolone with oral tapering.^11^

The coronary arteries became normal after six-weeks in our case, whereas a study from Lahore has revealed coronary artery involvement in 24.4% children with MISC, among them two third (70%) had persistent coronary changes on four week follow up 20% children had coronary changes after six months.^12^

## CONCLUSION

There should be high index of suspicion of Kawasaki disease while evaluating pediatric patients with COVID-19 infection so that timely interventions can be made to prevent adverse outcome of Kawasaki disease. More prospective studies are needed to evaluate the relationship between KD and Covid-19 infection.

### Authors’ Contributions:

**MT and RM** identified the case and prepared the manuscript.

**MS and RM** supervised all the steps in the preparation of the manuscript.

**MT, RM, and MS** take full responsibility for its clinical integrity and approve the final version of the manuscript.

### Patients consent:

Informed consent was obtained from parents.
